# Unsupervised Learning for Machinery Adaptive Fault Detection Using Wide-Deep Convolutional Autoencoder with Kernelized Attention Mechanism

**DOI:** 10.3390/s24248053

**Published:** 2024-12-17

**Authors:** Hao Yan, Xiangfeng Si, Jianqiang Liang, Jian Duan, Tielin Shi

**Affiliations:** 1 State Key Laboratory of Digital Manufacturing Equipment and Technology, Huazhong University of Science and Technology, Wuhan 430074, China; 2 School of Mechanical Science and Engineering, Huazhong University of Science and Technology, Wuhan 430074, China

**Keywords:** unsupervised feature learning, machinery fault detection, auto-encoder, kernelized attention, adaptive thresholding

## Abstract

Applying deep learning to unsupervised bearing fault diagnosis in complex industrial environments is challenging. Traditional fault detection methods rely on labeled data, which is costly and labor-intensive to obtain. This paper proposes a novel unsupervised approach, WDCAE-LKA, combining a wide kernel convolutional autoencoder (WDCAE) with a large kernel attention (LKA) mechanism to improve fault detection under unlabeled conditions, and the adaptive threshold module based on a multi-layer perceptron (MLP) dynamically adjusts thresholds, boosting model robustness in imbalanced scenarios. Experimental validation on two datasets (CWRU and a customized ball screw dataset) demonstrates that the proposed model outperforms both traditional and state-of-the-art methods. Notably, WDCAE-LKA achieved an average diagnostic accuracy of 90.29% in varying fault scenarios on the CWRU dataset and 72.89% in the customized ball screw dataset and showed remarkable robustness under imbalanced conditions; compared with advanced models, it shortens training time by 10–26% and improves average fault diagnosis accuracy by 5–10%. The results underscore the potential of the WDCAE-LKA model as a robust and effective solution for intelligent fault diagnosis in industrial applications.

## 1. Introduction

With the rapid development of Industry 4.0, modern industrial systems usher in digital transformation [1], requiring advanced fault detection to maintain safety and efficiency in production processes [2]. As an indispensable part of rotating machinery systems, bearings directly affect their performance and operation. However, subjected to complex loads and high-speed operation over extended periods, bearings are susceptible to wear and structural degradation, potentially compromising performance and operator safety [3,4]. Therefore, effective and timely fault detection of bearings is essential for maintaining industrial system stability [5].

Traditional bearing fault diagnosis methods depend on prior knowledge and conventional analysis techniques to derive diagnostic insights [6]. However, the unbalanced and unlabeled condition of bearings makes traditional methods, such as physical modeling, challenging to comprehensively address [7]. With advancements in artificial intelligence, data-driven fault diagnosis approaches are increasingly being adopted [8]. Unlike traditional techniques, data-driven methods do not require prior knowledge, allowing real-time fault detection based on sensor data [9]. Among these, deep learning-based intelligent fault diagnosis (IFD) demonstrates significant advantages in processing extensive long data and accurately assessing machinery health [10,11]. Kiakojouri et al. [12] proposed a hybrid technique combining improved cepstrum pre-whitening and high-pass filtering, demonstrating enhanced bearing fault diagnosis capabilities under various conditions, particularly in detecting incipient faults with low signal-to-noise ratios. Ding et al. [13] proposed an edge intelligence method for bearing fault diagnosis using a parameter-transplanted convolutional neural network (S-AlexNet), demonstrating high efficiency and low cost for embedded systems. Du et al. [14] introduced the Integrated Gradient-based Continuous Wavelet Transform (IG-CWT), an explainable deep learning method for bearing fault diagnosis that improves fault classification by selecting a key frequency range. Bertocco et al. [15] proposed a low-computational-cost machine learning model for roller bearing fault classification, designed for embedded microcontrollers, achieving efficient real-time fault detection using spectrogram-based preprocessing and a lightweight CNN architecture.

Yet, deep learning models generally require extensive, labeled, and balanced datasets; annotating extensive industrial datasets is costly and labor-intensive, where normal data are easily collected, resulting in data imbalances that compromise fault recognition accuracy [16]. Addressing the challenges of training deep learning models on unlabeled, imbalanced datasets is essential for effective fault diagnosis [17]. In practical equipment maintenance, bearings are often directly replaced when faulty, without necessitating precise fault localization or extent analysis [18]. Unsupervised deep learning methods, which do not require labeled samples, are well-suited to facilitate rapid anomaly detection in bearings [19]. These methods adhere to the basic process of intelligent fault diagnosis: data collection, feature extraction, and pattern recognition, with feature extraction playing a particularly critical role [20]. High-quality features not only reveal potential fault information but also distinguish diverse fault types [21].

Common unsupervised fault detection techniques based on feature extraction fall broadly into two categories: feature clustering and feature reconstruction. Clustering methods include K-means, K-medoids, DBSCAN, and HDBSCAN [22]. Feature reconstruction methods, such as Autoencoders (AE), are typical feature learning techniques that encode input data into lower-dimensional features and then decode to reconstruct the original data [23]. By modifying the basic AE, there are now many variants of AE models, such as DAE (Denoising Autoencoder), SAE (Sparse Autoencoder), convolutional autoencoders (CAE), and SAEs (stacked autoencoders) [24,25,26,27,28].To adapt to the unbalanced and unlabeled condition and improve model robustness and generalization ability, Zhang Z et al. [18] proposed the sparsity-optimized autoencoder (SOAE), demonstrating enhanced feature discrimination and robustness against noise. Zhang J et al. [29] proposed the modified denoising autoencoder with self-attention (MDAE-SAMB) for fault detection and representation learning for few-shot classification and unknown fault identification, achieving high accuracy and robustness. However, feature clustering methods often struggle with key feature extraction from long-term data, while feature reconstruction methods exhibit lower accuracy in addressing complex industrial environments. Therefore, there is a pressing need for an unsupervised fault diagnosis method that can efficiently extract data features and adapt to complex industrial environments for rapid and accurate bearing fault detection.

This study proposes a model based on a Wide-Kernel Convolutional Autoencoder (WDCAE) and a Large-Kernel Attention (LKA) mechanism, referred to as WDCAE-LKA, for advanced fault diagnosis of rolling bearings. The WDCAE module captures fine-grained features from raw vibration signals, while the LKA module improves the extraction of critical information. Additionally, a multi-layer perceptron (MLP)-based adaptive thresholding module enables dynamic threshold adjustments, enhancing model adaptability and reliability across diverse operational conditions. The main contributions of this paper are as follows:

(1) Powerful Feature Extraction Capability: The wide-kernel convolutional autoencoder, combined with the large-kernel attention mechanism, enhances fine-grained feature capture, significantly improving fault detection accuracy.

(2) Adaptive Threshold Adjustment Mechanism: The MLP-based adaptive thresholding module provides flexibility to adapt to the unbalanced condition, thereby improving diagnostic precision and reliability.

(3) Strong Adaptability to Changing Complex Industrial Environments: Experimental validation on two datasets demonstrates that the WDCAE-LKA model excels across both stable and variable operational conditions, underscoring its potential for broad industrial applications.

In conclusion, the WDCAE-LKA model combines advanced feature extraction with an adaptive threshold adjustment mechanism, offering a robust solution for fault diagnosis under complex conditions. The structure of the paper is as follows: Section 2 introduces the foundational principles of AE models and attention mechanisms; Section 3 details the WDCAE-LKA model; Section 4 presents experimental results and analysis; Section 5 concludes the paper.

## 2. Basic Theory

### 2.1. Standard Autoencoder

**Figure 1:**
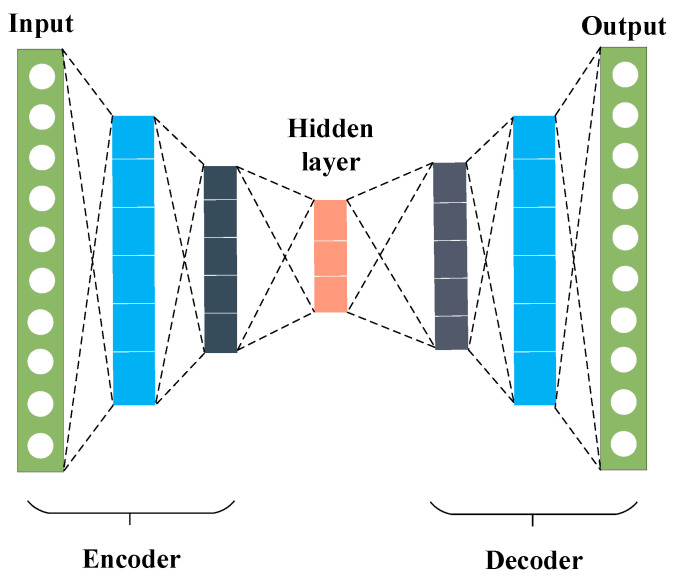
Network structure of AE.

The Autoencoder (AE) is a neural network-based technique used in unsupervised learning for dimensionality reduction and feature extraction. The basic structure of an AE, as shown in Figure 1, includes two primary processes: compression (encoding) and reconstruction (decoding), both implemented through neural networks. In its simplest form, an AE consists of a symmetrical three-layer architecture: an input layer, a hidden (or latent) layer, and an output layer. The input and output layers contain an equal number of neurons, ensuring that the AE attempts to reconstruct the input data accurately. When a sample $x\in R^{I}$ is introduced into the network, the encoder compresses this input, producing a feature vector $f\in R^{J}$ in the hidden layer. The decoder then reconstructs this feature vector, yielding an output vector $z\in R^{I}$. Here, I and J denote the dimensions of the input and hidden layers, respectively. The encoding and decoding processes are mathematically represented as follows:

(1) $$\sigma_{s}\left(t\right)=\frac{1}{1+\exp\left(-t\right)}$$

(2) $$f=\sigma_{s}\left(W_{e}x+{\;b}_{e}\right)$$

(3) $$z=\sigma_{s}\left(W_{d}f+b_{d}\right)$$

Here, $\sigma_{s}\left(t\right)$ represents the commonly used Sigmoid activation function. The weight matrices for the encoder and decoder are denoted by $W_{e}\in R^{I\times J}$ and $W_{D}\in R^{J\times I}$, respectively, while $b_{e}\in R^{J}$ and $b_{d}\in R^{I}$ are the bias vectors. The parameter set for the AE,$\left\{W_{e},W_{d},b_{e},b_{d}\right\}$, is optimized over the unlabeled training sample set {$x^{1},x^{2},\ldots ,x^{N}$} by minimizing the following cost function:

(4) $$\;\psi_{AE}\left(W_{e},W_{d},b_{e},b_{d}\right)=\frac{1}{2N}\sum_{n=1}^{N}{∥x^{n}-z^{n}∥}_{2}^{2}$$

After training, the optimized encoding function $\sigma_{s}\left(W_{e}^{*},b_{e}^{*}\right)$ can serve as a feature extractor for any input sample $r\in R^{I}$ [30].

The standard AE is widely applied due to its adaptive and robust feature learning capabilities, especially with non-sequential data. However, in complex time-series data, it only considers the current input, overlooking temporal correlations and thus limiting its ability to capture time-dependent patterns. To address this issue, this paper proposes a novel Wide-Kernel Convolutional Autoencoder (WDCAE) to improve feature extraction and adaptability. Through wide convolutional kernels, the WDCAE captures granular features during encoding, providing a clear advantage in reconstructing and classifying complex signals.

### 2.2. Self-Attention Mechanism

The self-attention mechanism is a core component of the Transformer architecture and enables sequence representation by computing correlations between distinct positions in a sequence. When handling long sequences, self-attention facilitates parallel computation of dependencies across positions, enhancing computational efficiency. The visualization of attention weights further aids in identifying focus regions within the model, thereby enhancing interpretability and providing insights into the decision-making process [31].

In the self-attention mechanism, the mapping of hidden layer neurons and their importance is derived through the following formula:

(5) $$Attention\left(Q,K,V\right)=softmax(\frac{{(QW}^{Q}){{(KW}^{K})}^{T}}{\sqrt{d_{k}}}){(VW}^{V})$$

(6) $$s=\phi \left(x_{e}^{c}\right)=\frac{1}{1+e^{-{(w}_{s}x_{e}^{c}+b_{s})}}$$

where $s=\left[s_{1},s_{2,}\ldots ,s_{b}\right]$, $w_{s}$ and $b_{s}$ are the weight matrix and bias vector in the self-attention layer, respectively, with ϕ as the sigmoid activation function. Here, *dk* represents the dimensionality of the key’s hidden representation, while $W^{K}$, $W^{Q}$, $W^{V}$ are the projection matrices for the query, key, and value, respectively, in a single attention head. For simplicity, in multi-head attention with *k* heads, we omit the notation for multiple sets of projection matrices in the equations below [31].

Traditional self-attention calculates attention scores via the dot product of the query and key, which can incur high computational costs and hinder performance in capturing complex nonlinear relationships. The Kernelized Attention module replaces the dot product with a kernel function, allowing the model to capture complex nonlinear dependencies more effectively, improving both computational efficiency and the ability to capture spatial correlations and long-range dependencies.

## 3. Proposed Intelligent Fault Diagnosis Method

**Figure 2:**
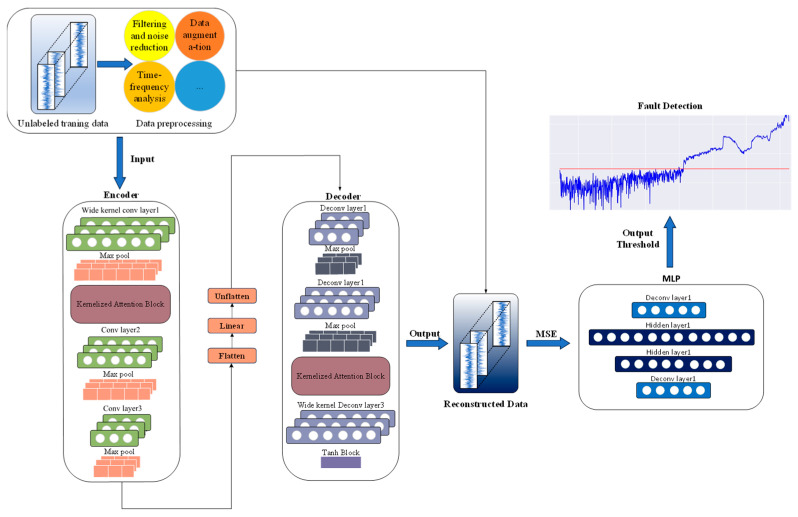
Overview structure of the proposed LKA-WDCAE.

Figure 2 illustrates the structure of the proposed LKA-WDCAE, which is composed of a main branch and multiple auxiliary branches. First, the bearing signal undergoes preprocessing through a wavelet denoising algorithm to minimize noise interference. Next, the designed LKA-WDCAE, which enhances the traditional three-layer AE structure, improves model performance while reducing parameter count, enabling accurate signal reconstruction. Finally, a Multi-Layer Perceptron (MLP) dynamically calculates an adaptive threshold based on the reconstruction error and signal features to identify fault signals under the unbalanced and unlabeled condition.

### 3.1. Large Kernelized Attention-Base Feature Extraction

The Large Kernelized Attention (LKA) module builds upon the standard attention mechanism by incorporating a kernel function transformation to refine the relationships between Query and Key. Define $Q\in R^{W\times L}$, $K\in R^{H\times L}$, and $V\in R^{m\times l}$ as the query, key, and value matrices, where W and H denote the sequence width and height, and L represents the feature dimensionality.

Initially, both the query and key matrices are transformed via a large kernel function $\phi \left(\cdot \right)$, resulting in the following mapped feature representations:

(7) $$\phi \left(Q\right)\in \;R^{W\times L},\;\phi \left(K\right)\in \;R^{H\times L}$$

**Figure 3:**
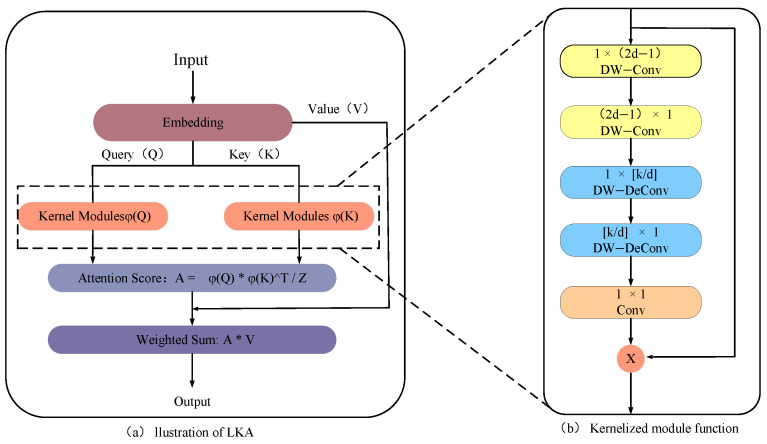
Structure of LKA.

As depicted in Figure 3a, the LKA module operates on an input feature map $F\in R^{C\times W\times H}$, where C represents the number of input channels, and d denotes the dilation rate. The output of the large kernel function is computed using Equations (8)–(11) as follows:

(8) $${\bar{Z}}^{C}=\sum_{H,W}W_{\left(2d-1\right)\times 1}^{C}*\;\left({\;\sum_{H,W}W_{1\times \left(2d-1\right)}^{C}*\;F}^{C}\right)$$

(9) $$Z^{C}=\sum_{H,W}W_{\left[\frac{k}{d}\right]\times 1}^{C}*\;\left(\sum_{H,W}W_{1\times \left[\frac{k}{d}\right]}^{C}\;*\;{\bar{Z}}^{C}\right)$$

(10) $$A^{C}=W_{1\times 1}\;*\;Z^{C}$$

(11) $${\bar{F}}^{C}=A^{C}\;⊗F^{C}$$

Here, ∗ and ⊗ denote convolution and Hadamard (element-wise) product, respectively. The term ${\bar{Z}}^{C}$ represents the output of depthwise convolutions of sizes (2d − 1) × 1 and 1 × (2d − 1) applied to the input feature map *F*, capturing localized spatial information and mitigating grid effects. $Z^{C}$ is derived by applying a depthwise convolution with kernel size $\left[\frac{k}{d}\right]$ to ${\bar{Z}}^{C}$. This output is then convolved with a 1 × 1 kernel to generate the attention map $A^{C}$. The final output of the LKA module, ${\bar{F}}^{C}$, is obtained as the Hadamard product of the attention map $A^{C}$ and the input feature map ${\bar{F}}^{C}$.

The attention score A is calculated as follows:

(12) $$A=\frac{\phi \left(Q\right)\cdot \;{\phi \left(K\right)}^{T}}{K}$$

where $\phi \left(Q\right)$ and $\phi \left(K\right)$ are the query and key matrices transformed by the kernel function module, respectively, and K is a normalization factor that ensures the sum of attention scores equals 1.

Next, the attention score A is used to perform a weighted summation over the value matrix V, resulting in the final output:

(13) Output=A⋅ V

This output is a weighted combination of nonlinear patterns captured by the kernel function, allowing the LKA module to handle complex input data patterns more effectively. Particularly suited for scenarios requiring higher-order feature extraction, the LKA design reduces computational complexity compared to the traditional self-attention mechanism. This efficiency enhancement makes LKA advantageous for industrial applications with rapid processing requirements.

### 3.2. Threshold Generation with LKA-WDCAE

The LKA-WDCAE model leverages unsupervised learning, which removes the requirement for labeled data in training. This model incorporates the Large Kernel Attention (LKA) mechanism in both encoding and decoding processes. In the encoding phase, the input signal is compressed into a low-dimensional latent space, while the decoder maps this latent representation back to reconstruct an output signal that closely resembles the original input.

During the encoding phase, the input data undergo feature extraction through convolutional layers and LKA layers, expressed as follows:

(14) $$C_{i}=f\left(\sum {W⨀x}_{i}+b_{i}\right)$$

(15) $$Z_{enc}=LKA\left(C_{i}\right)$$

where $C_{i}$ represents the feature map output from the convolutional layer, W is the convolution kernel, $x_{i}$ is the input feature map, $b_{i}$ is the bias term, and ⨀ denotes the convolution operation. $Z_{enc}$ is the encoded feature map output, and  LKA (⋅) denotes the application of the Large Kernel Attention module on the feature map. Through successive convolutional layers and LKA layers, the model incrementally extracts higher-level abstract features.

In the decoding phase, the encoder’s output is processed through transposed convolution layers with integrated LKA modules, represented by:

(16) $${\hat{C}}_{i}=f\left(\sum W^{T}⨀Z_{enc}+\hat{b_{i}}\right)$$

(17) $${\hat{X}}_{i}=LKA\left({\hat{C}}_{i}\right)$$

where $W^{T}$ is the transposed convolution kernel, $Z_{enc}$ is the decoder’s output, $\hat{b_{i}}$ is the bias term, and ${\hat{X}}_{i}$ is the output after the Large Kernel Attention layer. Key parameters such as stride and padding are used in the transposed convolution to control the spatial dimensions of the output feature map.

The model’s reconstruction loss is optimized using the Mean Squared Error (MSE) loss function, defined as:

(18) $$\mu_{mse}=\frac{1}{N}\;\sum_{i=1}^{N}{\left(X_{i}-{\hat{X}}_{i}\right)}^{2}$$

where $\mu_{mse}$ represents the final reconstruction error, $X_{i}$ is the original input data, ${\hat{X}}_{i}$ is the reconstructed output, and N is the sample size. By minimizing the MSE loss, the model iteratively optimizes its performance during training, thus improving reconstruction accuracy.

The integration of LKA modules significantly improves the model’s ability to process long-sequence signals, yielding higher reconstruction accuracy and establishing a robust foundation for applications involving complex datasets.

### 3.3. Threshold Adaptation with MLP

To improve fault detection under the unbalanced condition, an adaptive threshold prediction method utilizing a Multi-Layer Perceptron (MLP) is introduced. This section focuses on the structure and function of the MLP, explaining how it computes the adaptive threshold based on input features and integrates the reconstruction error from the LKA-WDCAE for fault detection.

The proposed method predicts the adaptive threshold by extracting various features from the reconstruction error of the LKA-WDCAE model, as explained in Section 3.2, and combines these with the time-frequency features of the original signal to form a comprehensive input dataset for training. The selected features, representing essential aspects of both the reconstructed and original signals, are listed in Table 1.

These extracted features provide a comprehensive view of both the reconstruction quality and statistical characteristics of the signal, enriching the input data for MLP module. The MLP receives the extracted feature vector $F=\left[f_{1},f_{2},\ldots ,f_{n}\right]$, which integrates features derived from both the reconstruction error of the LKA-WDCAE model and the vibration signal itself, to produce an adaptive threshold for effective fault detection. By dynamically adjusting the threshold based on these robust signal characteristics, the MLP aids in distinguishing between normal and fault conditions under diverse operating scenarios.

The MLP architecture is structured with two hidden layers, where each hidden layer employs the ReLU activation function, and the output layer uses a linear activation function to yield a continuous threshold value. The output formula of the MLP is as follows:

(19) $$\mu_{adaptive}=\int_{MLP}\left(F\right)=W_{L}\;\cdot \sigma \;\left(W_{L-1}\;\cdot \sigma \left(\ldots \sigma \left(W_{1}\cdot F+b_{1}\right)\ldots \right)+b_{L-1}\right)+\;b_{L}$$

where $\mu_{adaptive}\;$ is the final output adaptive threshold, $W_{i}$ represents the weight matrix of the i-th layer, $b_{i}\;$ is the bias vector of the i-th layer, σ is the ReLU activation function. L denotes the number of hidden layers. This formula describes how the input features are progressively transformed through each layer’s weights, biases, and activation functions in the MLP, ultimately generating an adaptive threshold tailored for reliable fault detection across varying conditions.

### 3.4. Combination Strategy Design

With the construction of the LKA-WDCAE module and MLP module complete, the next phase is to develop an integrated fault diagnosis decision system. This combination strategy involves the following key steps:

1. Data Collection and Denoising: Acquire unlabeled data from rolling bearings, applying noise reduction techniques to filter out industrial noise interference. This step is crucial for ensuring the input data quality, allowing the model to focus on meaningful signal features.
2. Feature Extraction and Reconstruction: Employ the LKA-WDCAE module to extract features from the denoised input data and perform accurate signal reconstruction. The module computes the reconstruction error of the dataset, which will be utilized in later stages for fault detection.
3. Threshold Generation: Using both the reconstruction error and original signal features obtained from the LKA-WDCAE module, train the MLP module to produce an adaptive threshold. This threshold acts as the criterion for distinguishing between normal and faulty signals.
4. Stability Assurance: Conduct repeated experiments and model training to ensure the stability and reliability of diagnostic results, making the system robust to various operational conditions.

The pseudo code for the proposed method is provided in Algorithm 1, and the detailed architectures of the LKA-WDCAE model components are summarized in Table 2. This combination strategy integrates feature extraction, threshold generation, and stability assurance to provide a comprehensive fault diagnosis framework tailored for complex industrial applications.
**Algorithm 1:** LKA-WDCAE# Training stage Input: unlabeled source datasets $x=\left[x_{1},x_{2},\ldots ,x_{m}\right]\in R^{1\times m}$ 1: for epoch = 1 to epochs do  2: Randomly sample source data from unlabeled datasets.  3. Extract features with the convolutional layer, using Equation (14) for  calculation.  4. Compute attention scores of feature maps with the large kernel  attention layer, using Equation (15).  5. Restore the signal through the deconvolution layer, calculated by Equation (16).  6. Calculate reconstruction error as output using Equation (18).  7. Use the output reconstruction error and original signal features as  input to the MLP.  8. Adaptively adjust the reconstruction error by Equation (19). 9.end for Return: The Adaptive Threshold $\mu_{adaptive}$. # Testing stage Input: Unseen target dataset $x^{\prime }$. Model: The Adaptive Threshold $\mu_{adaptive}$.. Output: Final diagnosis decisions.

## 4. Experimental Results and Discussion

To simulate real-world industrial scenarios, the proposed method was tested on two mechanical test rigs. The objective was to assess the performance of the LKA-WDCAE model and its individual components for fault detection under various conditions. The evaluation involved two main steps: initially, five variants of AE-based unsupervised methods were selected for comparison, and these methods served as a benchmark to measure the LKA-WDCAE model’s performance in terms of reconstruction accuracy and fault detection capability. Subsequently, ablation studies were conducted to verify the effectiveness of the different components of the method.

### 4.1. Dataset Description

(1) CWRU Public Bearing Dataset

**Figure 4:**
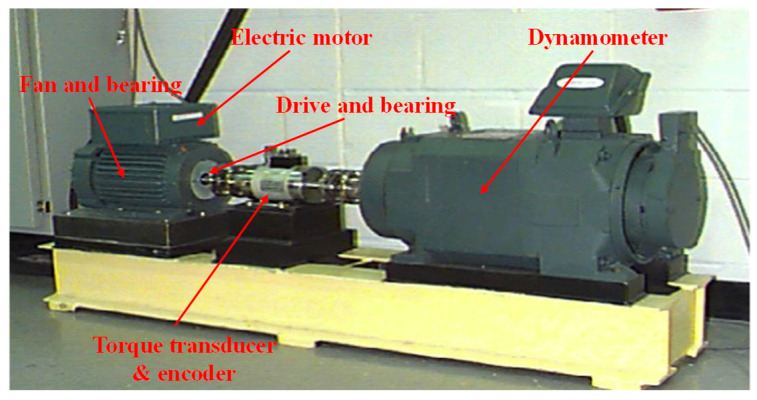
Schematic diagram of the experimental setup for the CWRU bearing dataset.

**Figure 5:**
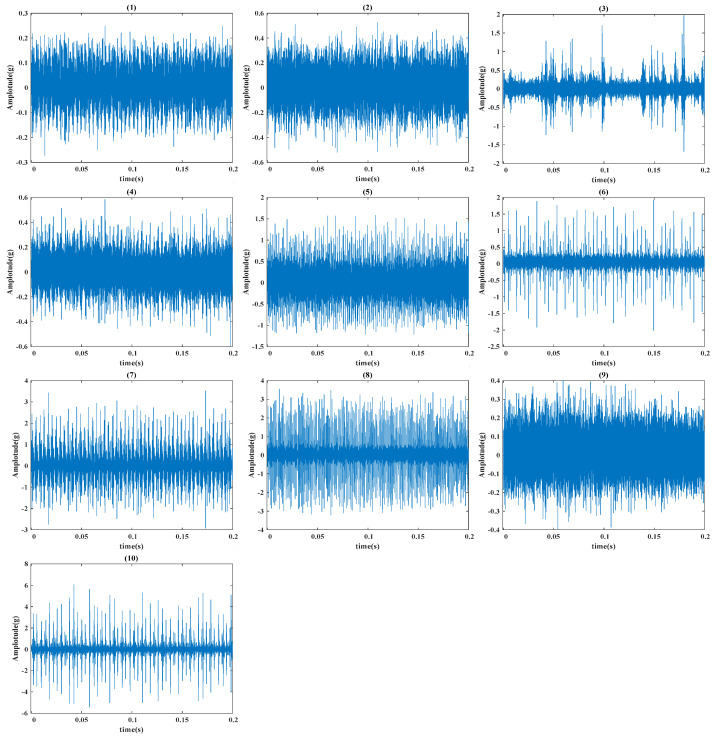
Raw vibration signals from the CWRU public dataset. (**1**) normal, (**2**) minor ball fault, (**3**) medium ball fault, (**4**) severe ball fault, (**5**) minor inner race fault, (**6**) medium inner race fault, (**7**) severe inner race fault, (**8**) minor outer race fault, (**9**) medium outer race fault, (**10**) severe outer race fault.

Figure 4 shows the schematic of the experimental setup from Case Western Reserve University Lab. The experimental setup mainly contains an induction motor, an accelerometer, testing bearings, and a loading motor. Vibration signals are collected by accelerometers [32]. Figure 5 displays the vibration waveforms for various fault conditions, including normal, rolling element fault, inner race fault, and outer race fault, with failure diameters of 0.178 mm, 0.356 mm, and 0.533 mm, respectively. Data are gathered from both the drive-end (DE) and fan-end (FE) bearings, at a rotational speed of 1772 rpm and a sampling frequency of 12 kHz. For each fault type, 200 samples are collected, with each sample comprising 1024 data points and 512 points overlapping with adjacent samples, resulting in 2000 total samples for each dataset. Table 3 provides a detailed description of the experimental data.

(2) Ball Screw System Bearing Fault Simulation Experimental Platform

**Figure 6:**
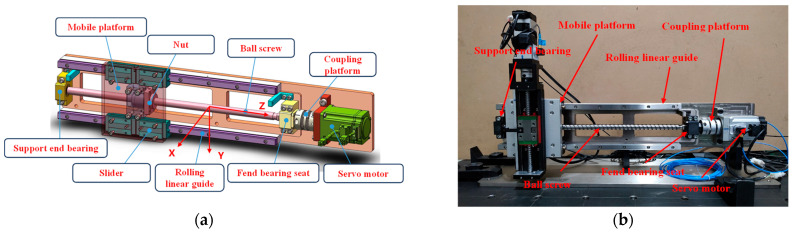
Schematic diagram of ball screw failure simulation experiment design: (**a**) ball screw system platform 3D model; (**b**) ball screw fault simulation experimental platform.

**Figure 7:**
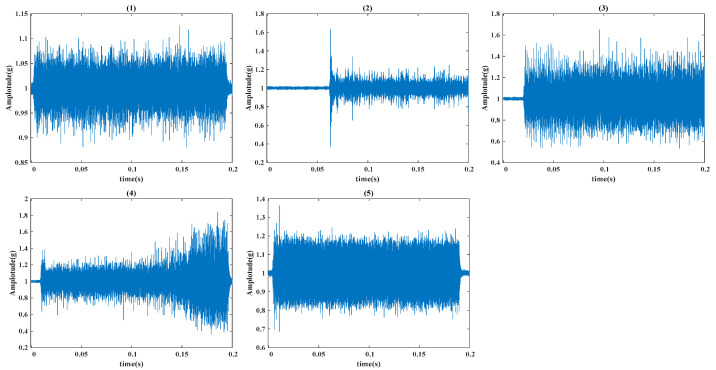
Original vibration signal from the self-built bearing fault experiment; (**1**) normal, (**2**) ball fault, (**3**) inner race fault, (**4**) outer race fault, (**5**) cage fault.

Figure 6 shows the 3D model of the ball screw system bearing failure experiment (a) and the actual failure simulation experiment platform (b). The platform mainly includes a mobile platform, rolling linear guide, support end bearing, fend bearing seat, ball screw, and a servo motor. This experiment uses two triaxial accelerometers to capture vibration signals from the platform. Additionally, three voltage sensors and three current sensors collect line voltage and current output from the servo motor. Signals are transmitted through National Instruments (NI) devices with a sampling frequency set to 10,240 Hz, while feedback from the motor driver, including command and actual position, speed, and output torque, is recorded at an approximate sampling frequency of 2000 Hz. Table 4 outlines the data collection arrangement. Using driver feedback data and timestamps, the motion time of each ball screw stroke is aligned with the sensor data to prepare samples for training and validating the intelligent diagnostic algorithm. Tests are conducted under various fault conditions, and Figure 7 illustrates the vibration signal waveforms for each fault, including inner race, outer race, rolling element, and cage faults, as well as normal conditions. The number of samples of each type in the original vibration signal is 500, and the length is 1024 data points.

### 4.2. Compared Approaches and Implementation Details

To comprehensively evaluate the performance of the proposed WDCAE-LKA model, we designed a series of seven fault detection tasks with varying proportions of normal and fault signals. These tasks simulate real-world industrial conditions where fault data availability may vary, reflecting scenarios from purely normal data to highly imbalanced datasets containing increasing proportions of fault signals. As shown in Table 5, the proportion of normal samples in the dataset used for model training in task1–task7 has decreased from 100% to 70%, and the proportion of faulty samples has increased from 0 to 30%. The diagnostic accuracy of a fault detection model will typically vary as the normal to fault ratio changes. Specifically, accuracy tends to decrease as the proportion of normal data decreases. This behavior is primarily due to the following factors: (1) The reconstructed models rely on sufficient normal data to establish baseline patterns for accurate anomaly detection; (2) at higher fault proportions, the overlapping characteristics of normal and fault signals become more pronounced, leading to increased reconstruction errors for both normal and faulty samples.

To evaluate the effectiveness of the proposed LKA-WDCAE model in addressing fault detection in unlabeled data, a comparative analysis was conducted involving several mainstream intelligent fault detection algorithms. Specifically, three basic AE model variants (M1–M3) were selected to benchmark the performance of the LKA-WDCAE. Additionally, two popular and advanced AE model variant algorithms (V1–V2) were tested to assess performance differences between the proposed method and state-of-the-art techniques (see Table 6 for details). Finally, an ablation study was performed using three model variants (A1–A3) to analyze the necessity of each component, and two model variants (B1 and B2) replaced the Large Kernel Attention Layer in A2 and A3 with a self-attention layer to analyze the advantages of the large core attention mechanism (see Table 7 for details).

All experiments were conducted on a Windows 11 64-bit system with an AMD Ryzen 7 5800H CPU (3.20 GHz) and an NVIDIA GeForce GTX 3060 GPU, utilizing the PyTorch (Version 2.0.0+cu118) framework. To minimize the influence of random variations, each experiment was repeated 10 times, with the average value taken as the final result. For consistency, all compared methods used the same backbone network as the proposed model to generate feature representations. The proposed method obtains the optimal values of learning rates, batch sizes, feature dimensions, kernel sizes, strides, and optimizers on the CWRU dataset through grid search (backbone network parameters, see Table 2). Each model was trained for 200 epochs, with a batch size of 32. The Adam optimizer was used with an initial learning rate of lr=0.001. Hyperparameters for the compared methods were set according to their respective original papers.

### 4.3. Diagnosis Results and Performance Analysis

(1) Comparison with unsupervised AE variants

**Figure 8:**
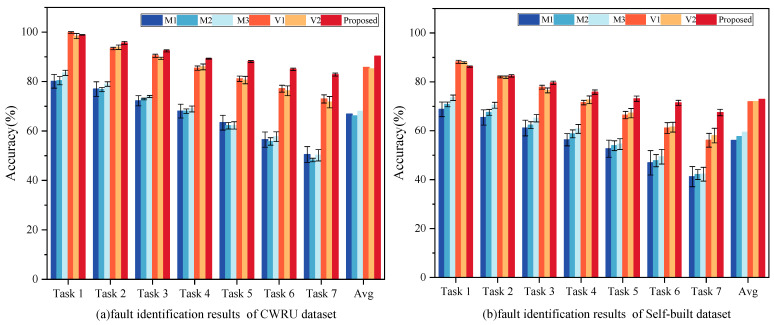
Experimental results of the proposed method and AE variant fault detection.

Three unsupervised baseline AE variants—DAE, VAE, and SAE (M1, M2, M3)—were selected as comparative baselines. Specifically, M1 enhances noise resistance by incorporating a denoising module in the bottleneck layer; M2 generates synthetic data by modeling the input data’s probability distribution, making it suitable for learning latent distributions in new samples; and M3 learns sparse features by optimizing sparsity constraints during the training lifecycle. Additionally, two widely recognized advanced unsupervised learning methods—MDAE-SAMB (V1) and SOAE (V2)—were also included as benchmark methods. V1 integrates multi-head attention mechanisms for improved feature discrimination within the DAE model, while V2 incorporates a sparsity optimization penalty term into the SAE model to enhance the sparsity of hidden layer feature distributions. The mean diagnostic accuracy outcomes from ten repeated experiments are presented in Figure 8, with specific result detailed in Table 8.

From Figure 8, four significant conclusions can be drawn: (1) The proposed LKA-WDCAE model, leveraging robust feature extraction capabilities, significantly outperforms the three basic AE variants across all diagnostic tasks, achieving the highest accuracy. This result suggests that LKA-WDCAE is well-suited to industrial applications involving unlabeled data. (2) Compared to other methods, the proposed model demonstrates notable performance gains under complex operational scenarios. This improvement is attributed to the reliance of other methods on reconstructing original signals for fault diagnosis, which does not account for the presence of fault signals within training samples. As the proportion of fault samples increases, the diagnostic accuracy of these models declines sharply, whereas LKA-WDCAE maintains high diagnostic accuracy due to the adaptive thresholding module’s integration. (3) In Task 1, both V1 and V2 outperform LKA-WDCAE, as these advanced methods possess specialized advantages in signal processing. According to Table 9, the proposed model improves training speed through the wide kernel design, resulting in slightly lower accuracy compared to V1 and V2 under certain conditions. However, as the proportion of fault signals increases, the diagnostic accuracy of LKA-WDCAE gradually becomes higher than that of V1 and V2. (4) Results across multiple experiments show that LKA-WDCAE has a significantly lower standard deviation compared to other models, signifying greater stability and robustness across complex industrial environments. This consistency in fault detection accuracy validates the model’s effectiveness in handling uncertainty and noise in industrial environments, underscoring its practical and applied significance in intelligent fault diagnosis systems.

(2) Effectiveness of Different Modules

**Figure 9:**
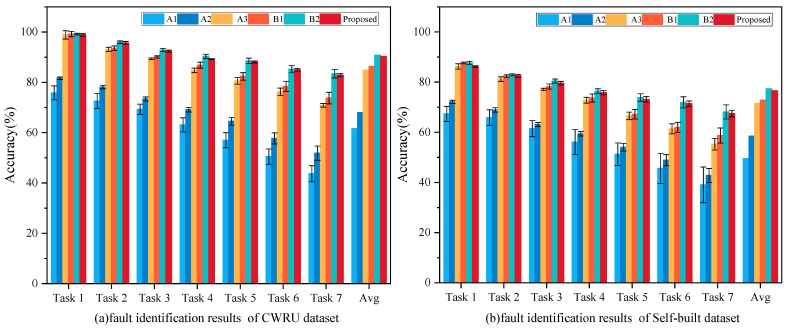
Experimental results of the proposed method and its variant fault detection.

An ablation study was conducted to evaluate the contribution of each model component by systematically removing individual components and observing the resulting performance changes. Five variants of the proposed method, outlined in Table 7, were used for this purpose. Figure 9 shows the diagnostic results of these variants, with specific results detailed in Table 10. By comparing the results of A3 with those of LKA-WDCAE, it is evident that the wide-kernel convolution and large-kernel attention layers demonstrably enhance feature extraction and reconstruction capabilities. By comparing B1 with A3, and B2 with LKA-WDCAE, we can find that the large core attention mechanism does reduce the prediction accuracy of the model compared with the self-attention mechanism, but it improves the speed of the model by 10–26% (see Table 11). The removal of the adaptive thresholding module resulted in a performance decline across all tasks except Task 1, underscoring the essential role of this module in adapting to unbalanced condition.

**Figure 10:**
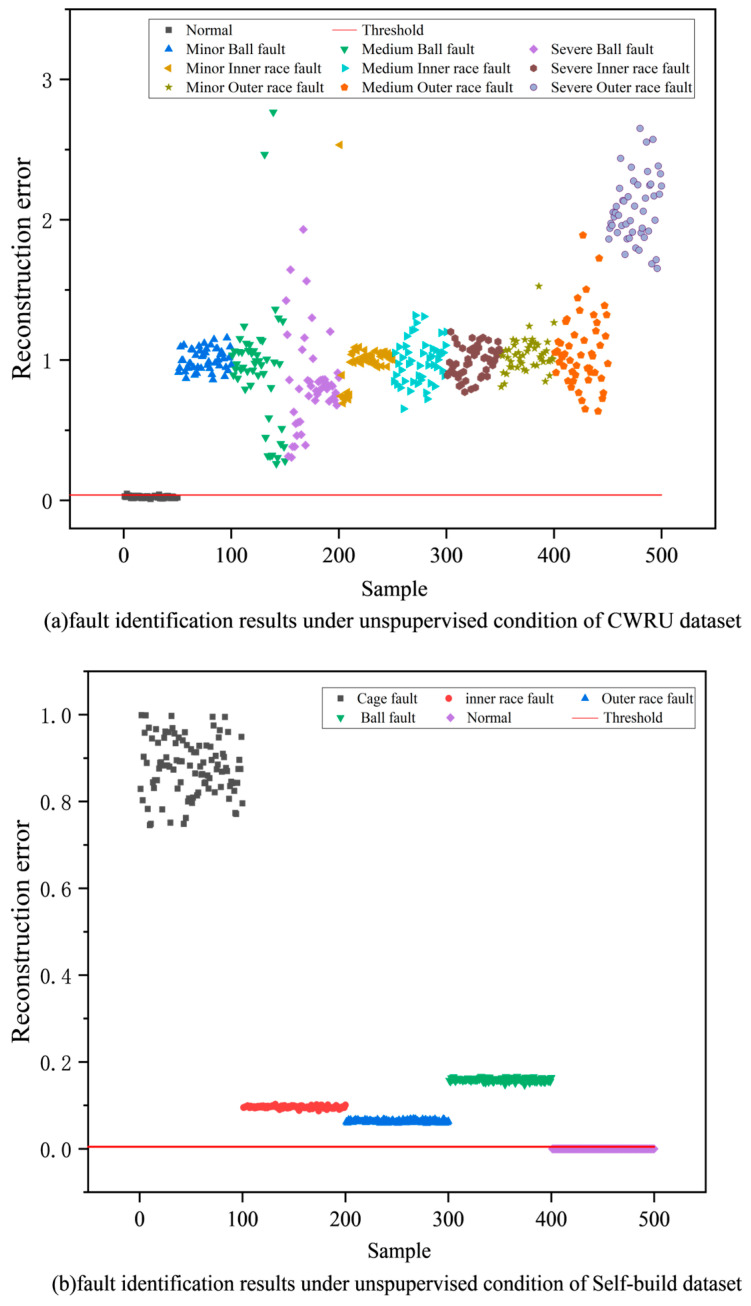
Visual fault detection verification results of two datasets in Task 5.

To provide a more intuitive representation of the model’s fault detection capabilities, the reconstruction error and detection threshold of test samples from Task 5 were plotted in two-dimensional space, as shown in Figure 10. The proposed method effectively distinguishes different types of signals and produces distinct clusters for each type.

This analysis underscores the essential role of each module in optimizing the overall performance of the proposed method. Specifically, the wide-kernel convolution and large-kernel attention mechanisms substantially enhance the model’s capacity for feature extraction and fault pattern reconstruction. Furthermore, the adaptive thresholding module proves indispensable for ensuring robustness and adaptability under unbalanced and unlabeled conditions.

## 5. Conclusions

In this study, we proposed a wide-kernel deep convolutional autoencoder model (LKA-WDCAE) for label-free fault diagnosis in industrial settings. The model innovatively integrates wide-kernel convolutional modules, large-kernel attention mechanisms, and an adaptive thresholding module to address the challenges of unlabeled, imbalanced, and noisy datasets. The model’s effectiveness was thoroughly validated through experiments on the CWRU dataset and a self-built dataset. Key findings include:

(1) The proposed model achieved superior diagnostic performance across varying fault scenarios. On the CWRU dataset, it attained an average accuracy of 90.29%, outperforming advanced methods such as MDAE-SAMB and SOAE by 4.54% and 5.13%, respectively. On the custom ball screw dataset, the model maintained high diagnostic accuracy, with a significant performance gain of 5–15% compared to baseline methods under imbalanced fault conditions.

(2) The adaptive thresholding module demonstrated robust adaptability, particularly in challenging scenarios with low fault signal proportions (e.g., Task 2 with only 5% fault signals, achieving 95.63% accuracy).

(3) In ablation studies, the integration of wide-kernel convolutions, large-kernel attention mechanisms, and the adaptive thresholding module resulted in a 15–20% improvement in accuracy compared to variants without these components.

(4) The proposed model exhibited a faster training speed compared to state-of-the-art methods, with a reduction in computation time of up to 20% on average across tasks.

The findings validate the feasibility of wide-kernel convolutions auto-encoder, large kernel attention mechanisms and MPL Adaptive module for label-free fault diagnosis, offering a viable solution for unsupervised fault diagnosis in industrial applications. The proposed approach demonstrates strong potential for practical implementation in real-world industrial environments.

Despite its promising performance, the proposed WDCAE-LKA model has certain limitations. First, though the wavelet denoising preprocessing step mitigates noise interference, extreme noise levels may still impact diagnostic accuracy. Secondly, the model’s adaptability to entirely unseen operating conditions requires additional validation. Future research will focus on addressing these limitations by incorporating domain adaptation techniques to enhance generalizability across unseen conditions, exploring lightweight attention mechanisms for real-time applications, and expanding the validation datasets to cover a broader range of industrial scenarios.

## Figures and Tables

**Table 1:** The extracted features and their formulas.

**Features of Error**	Features of Signal	Formula
Maximum	Maximum	$z_{max}=\max\left(z\right)$
Minimum	Minimum	$z_{min}=\min\left(z\right)$
Peak-to-Peak Value	Peak-to-Peak Value	$z_{p-p}=\max\left(z\right)-\min\left(z\right)$
Mean	Mean	$z_{mean}=\frac{1}{n}\sum_{i=1}^{n}z_{i}$
Variance	Variance	$z_{var}=\frac{1}{n}\sum_{i=1}^{n}{{(z}_{i}-\bar{z})}^{2}$
-	Root mean square	$z_{rms}=\sqrt{\frac{1}{n}\sum_{i=1}^{n}{z_{i}}^{2}}$
-	Skewness	$z_{skew}=E\left[{\left(\frac{z-\mu }{\sigma }\right)}^{3}\right]$
-	Kurtosis	$z_{kurt}=E\left[{\left(\frac{z-\mu }{\sigma }\right)}^{4}\right]$
-	Wavelet Coefficient	$E_{WT}=\sum_{i=1}^{N}wt_{\phi }^{2}\left(i\right)$/N

**Table 2:** Detailed structure parameters of the proposed method.

Module Name	Layer Type	Parameters	Operation
LKA-WDCAE	Convolution	Kernel 16–64 × 4	BN, ReLU
	Max-Pooling	Kernel 16–2 × 2	/
	Convolution	Kernel 32–3 × 1	BN, ReLU
	Large KernelAttention	256, head = 8	ReLU, Sigmoid
	Convolution	Kernel 64–3 × 1	BN, ReLU
	Max-Pooling	Kernel 64–2 × 2	Flatten
	Linear Layer1	64, 1024	
	Linear Layer2	1024, 64 × 128	UnFlatten
	ConvTranspose	Kernel 32–3 × 1	ReLU
	Large KernelAttention	256, head = 8	
	ConvTranspose	Kernel 16–3 × 1	ReLU
	ConvTranspose	Kernel 16–64 × 4	ReLU
MLP	Input Layer	Input Size: 14	
	Linear	Input: 14 Output: 128	BN
	Linear	Input: 128 Output: 64	ReLU
	Dropout	*p* = 0.2	
	Linear	Input: 64, Output: 32	ReLU
	Linear	Input: 32, Output: 1	Linear (None)

**Table 3:** Data distribution of CWRU bearing dataset.

Bearing State	Sensor	Diameter (mm)	Class Label	Data Length	Sample Number
Normal	DE & FE	-	Nor	1024	200
Rolling element fault		0.178	Ro07	1024	200
		0.356	Ro17	1024	200
		0.533	Ro21	1024	200
Inner ring fault		0.178	In07	1024	200
		0.356	In 17	1024	200
		0.533	In 21	1024	200
Outer ring fault		0.178	Ou07	1024	200
		0.356	Ou14	1024	200
		0.533	Ou21	1024	200

**Table 4:** Experimental data collection arrangement.

Signal Type	Sensor Model	Sensor Layout	Collection Device	Sampling Frequency
Vibration Acceleration Signal	PCB 356A16	1 attached magnetically to the fixed end bearing seat, another to the support end bearing seat or mobile platform	NI 9230	
Servo Information	—	—	Servo Driver Panasonic MBDLN25BE	~2000 Hz

**Table 5:** Unsupervised fault detection tasks for two datasets.

Task Name	Signal Proportion	Task Description
Task 1	100% normal signals, 0% fault signals	simulates a fault-free baseline scenario
Task 2	95% normal signals, 5% fault signals	simulate an early-stage fault scenario
Task 3	90% normal signals, 10% fault signals	simulate a moderate fault scenario
Task 4	85% normal signals, 15% fault signals	simulates more noticeable fault occurrences
Task 5	80% normal signals, 20% fault signals	simulates a challenging scenario
Task 6	75% normal signals, 25% fault signals	simulates real-world imbalanced datasets
Task 7	70% normal signals, 30% fault signals	simulates highly imbalanced datasets

**Table 6:** Variants of the five AE models.

Method	Description
M1	DAE, analogous to AE, with enhanced resistance to noisy signals.
M2	VAE, analogous to AE, offering advantages in generative capability, latent space continuity, generalizability, and interpretability.
M3	DAE, analogous to AE, with added sparsity constraints for learning more representative features.
V1	MDAE-SAMB [32], integrates attention mechanisms within neurons for efficient and precise fault detection.
V2	SOAE [14], includes two sparse optimization attributes for high-precision unsupervised fault detection.

**Table 7:** Ablation study.

Method	Wide Kernel Convolution Layer	Large Kernel Attention Layer	Self-Attention Layer	MLP Adaptive Thresholding Layer
A1	×	×	×	×
A2	√	×	×	×
A3	√	√	×	×
B1	√	×	√	×
B2	√	×	√	√
LKA-WDCAE	√	√	×	√

**Table 8:** Performance comparison of fault detection.

Task Name	M1	M2	M3	V1	V2	Proposed
CWRU-Task 1	80.36%	80.10%	83.52%	99.83%	98.39%	98.87%
CWRU-Task 2	76.74%	76.97%	78.99%	93.37%	93.89%	95.63%
CWRU-Task 3	72.96%	72.24%	73.96%	90.42%	89.42%	92.41%
CWRU-Task 4	67.99%	68.03%	68.91%	85.44%	85.91%	89.25%
CWRU-Task 5	62.13%	63.37%	62.24%	81.13%	80.60%	88.09%
CWRU-Task 6	55.76%	56.50%	57.68%	77.13%	76.28%	84.97%
CWRU-Task 7	48.24%	50.45%	50.18%	72.95%	71.66%	82.82%
CWRU-Avg	66.12%	66.95%	67.93%	85.75%	85.16%	90.29%
Ball screw-Task 1	68.78%	70.89%	73.52%	88.21%	86.91%	86.25%
Ball screw-Task 2	65.45%	67.54%	70.45%	82.04%	81.93%	82.47%
Ball screw-Task 3	61.12%	62.32%	65.12%	77.84%	77.52%	79.60%
Ball screw-Task 4	56.32%	58.75%	60.78%	71.54%	71.66%	75.82%
Ball screw-Task 5	52.67%	53.90%	54.54%	66.43%	67.21%	73.12%
Ball screw-Task 6	46.90%	47.78%	49.40%	61.13%	61.49%	71.43%
Ball screw-Task 7	41.23%	42.11%	42.25%	58.02%	53.45%	67.45%
Ball screw-Avg	56.07%	57.61%	59.43%	71.90%	72.25%	76.59%

**Table 9:** Computation training time (s) of three methods.

Task Name	V1	V2	LKA-WDCAE
CWRU-Task 1	98.68	113.13	96.78
Ball screw-Task 1	104.42	115.10	101.93

**Table 10:** Performance comparison of the variants and LKA-WDCAE.

Task Name	A1	A2	A3	B1	B2	LKA-WDCAE
CWRU-Task 1	75.82%	81.63%	98.87%	99.21%	99.21%	98.87%
CWRU-Task 2	72.58%	78.12%	93.12%	93.54%	95.97%	95.63%
CWRU-Task 3	69.30%	73.39%	89.37%	90.12%	92.84%	92.41%
CWRU-Task 4	63.12%	69.14%	84.76%	86.85%	90.34%	89.25%
CWRU-Task 5	56.99%	64.51%	80.58%	82.37%	88.53%	88.09%
CWRU-Task 6	50.45%	57.72%	76.23%	78.43%	85.23%	84.97%
CWRU-Task 7	43.67%	51.86%	70.88%	73.76%	83.45%	82.82%
CWRU-Avg	61.57%	68.04%	84.83%	86.32%	90.79%	90.29%
Ball screw-Task 1	67.34%	72.34%	86.25%	87.64%	87.64%	86.25%
Ball screw-Task 2	65.88%	68.92%	81.23%	82.43%	82.91%	82.47%
Ball screw-Task 3	61.45%	63.11%	77.11%	78.25%	80.47%	79.60%
Ball screw-Task 4	56.10%	59.45%	72.78%	73.68%	76.42%	75.82%
Ball screw-Task 5	51.25%	54.03%	66.54%	67.19%	73.85%	73.12%
Ball screw-Task 6	45.63%	48.89%	61.40%	61.96%	71.96%	71.43%
Ball screw-Task 7	39.05%	42.76%	55.25%	58.73%	68.13%	67.45%
Ball screw-Avg	49.53%	58.50%	71.51%	72.84%	77.34%	76.59%

**Table 11:** Computation training time (s) of five methods.

Task Name	A1	A2	A3	B1	B2	LKA-WDCAE
CWRU-Task 1	65.14	82.35	91.28	107.81	122.32	96.78
Ball screw-Task 1	72.56	86.23	94.72	112.15	128.47	101.93

## Data Availability

Data are contained within the article.
